# A Comprehensive Review on Potential In Silico Screened Herbal Bioactive Compounds and Host Targets in the Cardiovascular Disease Therapy

**DOI:** 10.1155/2024/2023620

**Published:** 2024-10-29

**Authors:** Elham Zarenezhad, Ali Tareq Hadi, Ensieh Nournia, Sadegh Rostamnia, Abdolmajid Ghasemian

**Affiliations:** ^1^Noncommunicable Diseases Research Center, Fasa University of Medical Sciences, Fasa, Iran; ^2^Womens Obstetrics & Gynecology Hospital, Ministry of Health, Al Samawah, Iraq; ^3^Cardiology Department, Hamadan University of Medical Sciences, Hamedan, Iran; ^4^Organic and Nano Group, Department of Chemistry, Iran University of Science and Technology, PO Box 16846-13114, Tehran, Iran

**Keywords:** cardiovascular disease, computational studies, natural bioactive compounds, therapy

## Abstract

Herbal medicines (HMs) have deciphered indispensable therapeutic effects against cardiovascular disease (CVD) (the predominant cause of death worldwide). The conventional CVD therapy approaches have not been efficient and need alternative medicines. The objective of this study was a review of herbal bioactive compound efficacy for CVD therapy based on computational and in silico studies. HM bioactive compounds with potential anti-CVD traits include campesterol, naringenin, quercetin, stigmasterol, tanshinaldehyde, Bryophyllin A, Bryophyllin B, beta-sitosterol, punicalagin, butein, eriodyctiol, butin, luteolin, and kaempferol discovered using computational studies. Some of the bioactive compounds have exhibited therapeutic effects, as followed by in vitro (tanshinaldehyde, punicalagin, butein, eriodyctiol, and butin), in vivo (gallogen, luteolin, chebulic acid, butein, eriodyctiol, and butin), and clinical trials (quercetin, campesterol, and naringenin). The main mechanisms of action of bioactive compounds for CVD healing include cell signaling and inhibition of inflammation and oxidative stress, decrease of lipid accumulation, and regulation of metabolism and immune cells. Further experimental studies are required to verify the anti-CVD effects of herbal bioactive compounds and their pharmacokinetic/pharmacodynamic features.

## 1. Introduction

**Figure 1:**
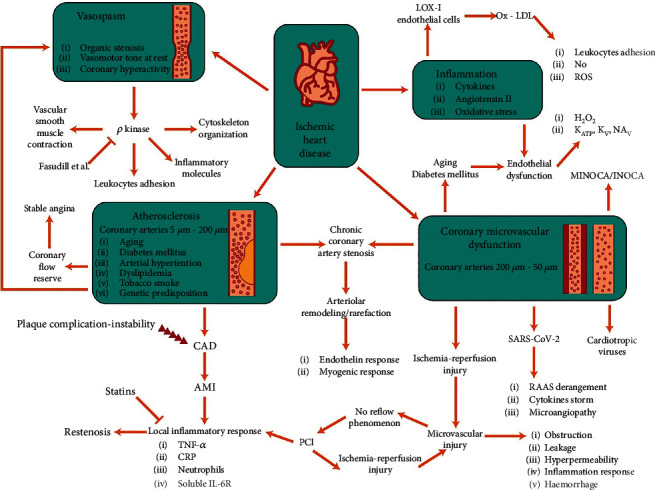
The pathophysiology of CVD/CHD: this occurs following the disturbance in the myocardial energy state and blood flow balance. LOX1, lectin-like oxidized low-density lipoprotein receptor-1; LDL, low-density lipoprotein; NO, nitric oxide; ROS, reactive oxygen species; MINOCA/INOCA, myocardial infarction/myocardial ischemia with no obstructive coronary artery disease; SARS-COV-2, severe acute respiratory syndrome coronavirus 2; CAD, coronary artery disease; AMI, acute myocardial infarction; PCI: percutaneous coronary intervention.

Cardiovascular diseases (CVDs) are among the leading causes of death worldwide, mainly due to stroke and heart attacks [1, 2]. The CVD incidence is disproportionally increasing globally, particularly in low-income and developing countries. Oxidative stress is a cause of numerous diseases, and hence its targeting is a promising approach [3]. The excess production of reactive oxygen species (ROS), including free radicals and nonradical molecules, leads to oxidative stress that causes endothelial dysfunction, and inflammation occurs mainly through the NF-*κ*B pathway [4, 5]. Hypertension and atherosclerosis have also been associated with CVD occurrence and development. Owing to the impossibility of adequate therapy, special attention to the disease is an unmet requirement [6]. CVD has a high burden globally in terms of personal quality of life and social and economic influences; besides, associated drug efficacies are poor [7]. Ischemic heart disease has also had a high and sharply increased fatality rate during the recent two decades [8]. In addition, difficult adherence to drug consumption by patients is due to adverse effects of chemical drugs and lack of affordability in low- or middle-income countries. Coronary heart and peripheral arterial diseases (CHD and PAD, respectively) are the main forms of CVD worldwide [9]. The CHD also occurs due to the obstruction of coronary arteries known as atherosclerosis. Cell signaling and molecular messengers are pivotal for the function of cardiac cells [10]. The pathophysiology of CVD/CHD has been depicted in Figure 1 [11].

The drawbacks in CVD/CHD treatment and control mainly include side effects of medications, cost of treatment, limited effectiveness of medications, lifestyle changes, long-term management, psychological impact, coexisting conditions, invasive procedures, limited access to healthcare, and genetic or familial factors [12–15]. On the other hand, developing novel drugs using experimental studies is a long time process that needs high costs and can be reduced using computational approaches [16–18].

Natural or herbal medicines (HMs) have been used for thousands of years in spite of the paucity of data on bioactive ingredients and mechanisms of action [19]. Fortunately, in silico and computational studies have contributed to a high degree in discovering and appreciating potential bioactive compounds and their targets (drug–target interactions) to treat various diseases such as CVD [17, 18, 20, 21]. HM ingredients are nontoxic, inexpensive/affordable, available, and mostly do not exert side effects [22]. They act through various mechanisms such as antioxidative, anti-inflammatory, antithrombotic, vasodilatory, and hypolipidemic effects [23].

## 2. Mechanisms of CVD Development

It has been demonstrated that inflammation plays a central role in the CVD progress in all stages of the disease via cholesterol accumulation, cell dysfunction, foam cell, plaque formation, and massive cytokine or interleukin (IL) release [24, 25]. The activation of vascular cells following the recruitment of immune cells such as T cells and macrophages is an important cause of atherosclerosis. The production of proinflammatory cytokines by these cells exacerbates the vascular lesions [26]. Excess nutrition in infants causing fast overweight is considered a risk factor for the development of CVD. ROS has an important role in the progression of CVD, particularly among diabetic patients. Another mechanism of vascular complications backs to Type 2 diabetes mellitus, in which chronic glycemia leads to the accumulation of advanced glycation end products (AGEs) [27]. Coronary embolism, coronary artery stenosis, microcirculatory disorders, vascular endothelial dysfunction, and vasospasm are major pathophysiology mechanisms of ischemic cardiomyopathy (ICM) [28].

## 3. Risk Factors of CVD

Among a variety of risk factors for CVD, age, genetic background, gender (permanent factors), obesity and dyslipidemia or overweight, diabetes mellitus, physical inactivity, smoking, and arterial hypertension (modifiable factors) have mainly participated [29, 30]. The reinforcement effects of these factors are also considerable. In addition, platelets contribute as a key factor in hemostasis and thrombosis, causing atherosclerosis and coronary artery disease (CAD). Other changeable risk factors include nutritional behavior or diet, and antioxidant containment and lifestyle affect the status of CVD or coronary events. Inflammatory conditions may also predispose to CVD. Hypertension, low education, and high cholesterol levels are other risk factors for CVD (China). It has been revealed that dyslipidemia, obesity, hypertension, physical inactivity, and malnutrition are major risk factors for CVD [31].

## 4. Common CVD Therapies and Side Effects

Common therapeutic approaches for CVD include surgery, medication, and cardiac rehabilitation [32, 33]. Angioplasty by inserting a balloon-like device is associated with bleeding, infection, and damage to blood vessels [34]. Coronary artery bypass grafting (CABG) may also be accompanied by infection, bleeding, and complications from anesthesia [35].

Statins mitigate cholesterol levels but may have some side effects such as muscle pain, liver damage, and an increased risk of diabetes [36]. Antiplatelet agents (e.g., aspirin and clopidogrel) are associated with gastrointestinal bleeding and easy bruising [37]. Beta-blockers regulate hypertension, and side effects include fatigue, dizziness, and sexual dysfunction [38]. ACE inhibitors and angiotensin receptor blockers (ARBs) are vasodilators and lower blood pressure. Their side effects include dry cough, low blood pressure, and kidney problems. Pacemaker implantation for heart rhythm regulation may also be followed by infection, bleeding, and device-related complications [39, 40].

## 5. HMs for the Treatment of CVD

HMs and traditional medicine systems have been used for centuries to address various health conditions such as CVD [41]. Several HMs and their bioactive compounds have demonstrated healing effects against CVD [42]. A recent systematic review has introduced *Zingiber officinale*, *Camellia sinensis*, *Curcuma longa*, *Crataegus* spp., *Allium sativum*, *Gentiana lutea*, *Bombax ceiba*, *Gynostemma pentaphyllum*, *Gongronema latifolium*, *Celosia argentea*, *Ginkgo biloba*, ginseng, and *Moringa oleifera* as potential HMs with this regard [41]. These HM potential benefits include managing risk factors and symptoms of the disease. One of their mechanisms is targeting oxidative stress [3]. Garlic (*Allium sativum*) has outlined potential to cure hypertension, reduce cholesterol levels, and inhibit platelet aggregation for managing CVD risk factors [43, 44]. Hawthorn (*Crataegus*) has also improved symptoms of chronic heart failure, angina, hypertension, and lipid profile [45]. Ginger (*Zingiber officinale*) has also been utilized for many years for disease healing such as CVD symptoms [46]. Traditional use of turmeric (*Curcuma longa* L.) and its main compound curcumin has also been documented in previous years, particularly in chronic diseases [47–49]. *Ginkgo biloba* flavonoids have been utilized for CVD treatment and protective effects [50, 51]. Green tea (*Camellia sinensis*) has also been used as a remedy for obesity, diabetes, and CVD [52, 53]. A review study revealed that *Phaseolus vulgaris* L., a common globally used nutritious legume or bean, has been associated with lowering CVD risk via various mechanisms. Its responsible bioactive compounds include fatty acids, phenolic acids, and flavonoids [54]. Various herbs in traditional Chinese medicine (TCM) have exhibited therapeutic effects for CVD mainly via anti-inflammatory effects [55, 56]. HMs also provoke gut microbiota to improve CVD health conditions [57]. HMs and natural compounds also act via oxidative stress, protein expression inhibition, regulation of matrix proteins, calcium levels, reduction in vascular smooth muscle cell (VSMC) proliferation and migration, improvement of mitochondrial functions, nitric oxide (NO) reduction, inhibition of apoptosis, and angiogenesis of endothelium [58]. HMs also reduce cardiac hypertrophy and improve the ATP-sensitive potassium (K_ATP_) channel function of myocardial cells [59, 60]. These agents also inhibit NO production in monocytes and macrophages and activate estrogen receptors and PPAR-*α* [61]. For instance, *Cinnamomum camphora* Linn has deciphered various health effects, such as heart tonic and function, thanks to numerous bioactive compounds [62]. Furthermore, protein targets have been demonstrated using bioinformatics studies [63]. A study has demonstrated the therapeutic effects of colchicine on CVD, and its mechanism of action included anti-inflammatory action, reducing proinflammatory cytokines, and inhibition of NOD-Like Receptor Protein 3 (NLRP3) inflammasome and NF-*κ*B signaling [64]. Another study revealed the antioxidative, antihypertensive, and cardioprotective potential of lycopene by decreasing the low-density lipoprotein (LDL) and improving high-density lipoprotein (HDL) [65].

## 6. Machine Learning (ML) Techniques

The most popular ML algorithms include RF, NB, AB, and KNN. These algorithms are used for classification and regression tasks [66], enhance accuracy, and improve overall predictive performance [67] and handling classification tasks [68, 69]. These algorithms are a type of artificial intelligence (AI) that enables computers to learn from and make decisions or predictions based on data. The patterns in data are identified by these algorithms using statistical techniques which also adjust their models accordingly, resulting in the improvement of their performance over time. Furthermore, these algorithms have applications for a wide range of purposes such as image and speech recognition, recommendation systems, and fraud detection [70]. Regarding CVD, ML has improved the disease prediction and recognition of its stages which can facilitate early intervention [20]. Additionally, the ability to take differences in data and accuracy in prediction can be achieved by a collection of several models [21, 71]. ML various algorithms are promising in the rapid and efficient drug discovery and its acceleration such as the traditional hit identification process. In addition, structural evaluation and biological activities of bioactive compounds and their metabolism can be assessed and predicted using ML [72–74]. The exploration of the chemical space of natural products is a crucial aspect of ML application [75–77].

## 7. Bioinformatics Study Efficacy for Finding Novel Drugs

Lead or potential therapeutic bioactive compound discovery strategy using computational studies is promising in terms of time and cost savings, accuracy, validity, reliability, and reproducibility [78]. It is based on the structure of the target protein and active site or any residues of interest to control its function. In recent years, various diseases have been targeted using in silico computational studies. Some of the findings have been verified as followed by in vitro, in vivo, and even clinical trial studies, opening avenues for efficient and safe ailment treatment using natural bioactive compounds. The significance of binding energy can be affected by a number of variables, and absolute values may differ based on the experimental conditions or the system being investigated [71, 79, 80]. Furthermore, network pharmacology has the potential to discover numerous compounds targeting various diseases. A support vector machine has been used to determine temperament (Mizaj) and uterine dystemperament (Su'-I-Mizaj Al-Rahim) roles in the treatment of abnormal vaginal discharge [81].

## 8. Experimental Studies

The experimental evaluation of these bioactive compounds has included in vitro, in vivo, and clinical trials. Huo-Tan-Chu-Shi Decoction (HTCSD) (mainly with magnoflorine and luteolin) including crude herbs increased the MAPK phosphorylation in the animal model via western blot analysis [82]. Punicalagin could increase the expression of NR1H3 (LXR), MSR1 (SRA), and CD36 genes in THP1 cells [26]. Regarding clinical trials, the campesterol/cholesterol ratio has increased among CVD patients. Therefore, more in-depth verification is needed to uncover their effects in lowering cholesterol [83]. Naringenin bioavailability and pharmacokinetics are limited; however, data supporting its protective roles in CVD and clinical trials are needed [84]. A study revealed the physical improvement using quercetin (1250 mg/day, 3 days/week) for 3 weeks [85]. Moreover, quercetin 500 mg bid reduced inflammatory factors and adipose tissue [86]. This concentration for 8 weeks also decreased oxidative stress and proinflammatory cytokines (IL-6 and tumor necrosis factor [TNF]-*α*) [87]. Quercetin has also exerted antiblood pressure or hypertension effects [88]. A randomized clinical trial of punicalagin for 20 weeks reduced the LDL levels significantly and improved dyslipidemia in adults [89]. Further clinical trials are required for the accurate determination of the CVD protective effects of potential HMs bioactive compounds found by bioinformatics studies [89].

## 9. Natural Bioactive Compounds for the Treatment of CVD Using Bioinformatics Studies

### 9.1. Molecular Docking Studies

In an in silico study, 4,926,615 bioactive compounds (using an e-pharmacophore model) targeting phosphodiesterase (PDE)5A and PDE3A with docking scores (DSs) of −12.10 and −10.01 kcal/moL, respectively, deciphered potential therapeutic effects against CVD [90]. Renin inhibitors were screened using docking assays, and potential bioactive compounds DS (kcal/moL) included RJC03502 (−9.275), SEW04046 (−8.574), RJC03497 (−8.232), JFD03561 (−7.701), JFD01243 (−7.477), and JFD03558 (−7.419) [91]. Bioactive compounds of *Bulbus allii Macrostemi* and *Trichosanthes kirilowii* Maxim herbs included *β*-sitosterol, naringenin, prostaglandin B1, and quercetin to treat CHD. Molecular targets of these bioactive compounds included prostaglandin-endoperoxide synthase (PTGS), Nitric Oxide Synthase 3 (NOS3), tumor necrosis factor (TNF), IL-6, B-Cell Lymphoma 2 (BCL2), and Vascular Cell Adhesion Molecule 1 (VCAM1) proteins. Highest DS (kcal/moL) rates included quercetin-PTGS2 (−9.7), naringenin-PTGS2 (−9.4), quercetin-NOS3 (−8.6), and *β*-sitosterol-TNF (−8.3) [92]. A TCM herbal complex of bioactive compounds was determined using data mining and network pharmacology to treat CHD acting via the calcium signaling pathway and neuroactive ligand-receptor binding. Target compounds included vascular endothelial growth factor A (VEGFA)-ginsenoside f2, Mitogen-Activated Protein Kinase 1 (MAPK1)-sitosterol, IL-6-stigmasterol, TNF-7-methoxy-2-methyl isoflavone, and PTGS2-fumarine as five drug targets towards the CHD treatment [93]. In a study, Qishen Yiqi Pills (QSYQP) anti-CVD bioactive compound targets (DS, kcal/moL) included tanshinaldehyde-PIK3CA (phosphatidylinositol-4,5-bisphosphate 3-kinase catalytic subunit alpha) (−11.1), kaempferol-SRC (−9.5), quercetin-EGFR (epidermal growth factor receptor) (−9.1), and tanshinaldehyde-PIK3R1 (Phosphoinositide-3-Kinase Regulatory Subunit 1) (−7.6). Their results were verified by western blotting, and the expression of FoxO1, DIAPH1 (Diaphanous Homolog 1), and ACTC1 (Actin Alpha Cardiac Muscle 1) genes was significantly decreased during hypoxia, while protein levels were significantly increased by supplementation of QSYQP [94]. It was unraveled that adenosine monophosphate-activated protein kinase (AMPK) and inducible nitric oxide (iNOS) targeting by *Bryophyllum pinnatum* Bryophyllin B bioactive compound (DS = −9.9) could treat atherosclerosis. Bryophyllin B had a significantly stronger and more stable interaction with the AMPK [95]. Higher stability of binding infers a better therapeutic effect of the compound as the binding is more stable. Moreover, structural stability was demonstrated. Baihe decoction *β*-sitosterol and quercetin bioactive compounds targeting MAPK1 and AKT1 (alpha serine/threonine-protein kinase) enzymes had promising effects against CHD. DS of *β*‐sitosterol‐MAPK1 = −7.5, *β*‐sitosterol‐AKT1 = −7.8, quercetin‐MAPKI = −6.5, and quercetin‐AKT1 = −6.5. Network pharmacology also found targets including FOS, ESR1 (Estrogen Receptor 1), EGFR, CXCL8 (C-X-C Motif Chemokine Ligand 8), IL-6, MAPK14, RELA (recombinant human transcription factor p65), MAPK1, AKT1, and Jun proteins. Punicalagin bioactive compound as a main ingredient of pomegranate peel was found to interact with cluster of differentiation 36 (CD36, DS = −9.3 kcal/moL), followed by toll-like receptor (TLR) 4, TRAF1 (tumor necrosis factor receptor–associated factor) (each DS = −9.0 kcal/moL), and peroxisome proliferator–activated receptors (PPAR-*γ*) (−8.7 kcal/moL). Their study findings were followed by gene expression using microarray for confirmation. Thereby, punicalagin could exert anti-inflammatory, lipid metabolism, and cell proliferation regulatory effects to prevent atherosclerosis [26]. Xintong Granule medicinal herb has demonstrated anti-CHD via targeting the Phosphoinositide-3-Kinase-Protein Kinase B (PI3K-AKT), MAPK, and JAK/STAT (Janus kinase/signal transducer and activator of transcription) signaling pathways. The main bioactive compounds included luteolin, kaempferol, fisetin (targeting TNF), naringenin, and nobiletin (targeting MAPK1) [96].

### 9.2. Network Pharmacology Studies

Network pharmacology is based on multiple drugs and target interactions in various diseases which systematically uncover molecular mechanisms. HTCSD is a common TCM that was deciphered with promising anti-CHD effects using network pharmacology and experimental studies. The main ingredients included luteolin, 13-hydroxyberberine, menisperine, and magnoflorine, targeting AKT1, EGFR, MAPK1, and SRC. Experimental confirmation of results was demonstrated via the phosphorylation of MAPK attributed to the luteolin and magnoflorine ingredients [82]. Molecular docking and network pharmacology revealed that *Dalbergia odorifera* (DO) contain three main bioactive compounds including butin, butein, and eriodyctiol targeting the AKT1 protein to treat ischemic stroke (Table 1) [99]. Further cell culture and animal studies demonstrated that three compounds conferred PC12 cell viability at higher doses. Western blot analysis exhibited AKT phosphorylation compared to the control (*p* < 0.01). *Tripterygium wilfordii* Hook F is a TCM containing various bioactive compounds. Network pharmacology uncovered that MAPK1, APP (amyloid beta precursor protein), AKT, PIK3CA, and TP53 (Tumor Protein 53) were the main targets for treating the CVD. The main bioactive compounds included triptofordin B2, hypodiolide A, celaxanthin, and celallocinnine [101]. A study deciphered that *Panax notoginseng* saponins prevent CHD and protect the endothelial cells from H_2_O via the inhibition of apoptosis and expression of the VEGFA mRNA expression. Additionally, IL-17, relaxin, shear stress pathway, and VEGF signaling pathways were affected. Notoginsenoside R1, ginsenoside Rg1, ginsenoside Re, ginsenoside Rb1, and ginsenoside Rd were major bioactive compounds. Further experimental study outlined altered expression of VEGFA and BCL2A1 (Bcl-2-related protein A1) genes [102]. Gualou Xiebai decoction (GLXB) was assessed for its bioactive compounds and anti-CHD effects and contained 53 ingredients. 2,3,4,9-Tetrahydro-1H-pyrido[3,4-b]indole-3-carboxylic acid and macrostemonosides S and B targeted the HRAS (GTPase HRas) pathway. Other protein targets included CDK2, HSP90AA1, SRC, ESR1, TAP1 (Transporter Associated with Antigen Processing 1), PTPA1 (Protein-Tyrosine Phosphatase 1), MAP2K1, AR (androgen receptor), NR3C1 (glucocorticoid receptor gene), TTR (transthyretin), MAPK14, and PPP1CC (Protein Phosphatase 1 catalytic subunit gamma) [103]. Mahai capsules contained 57 bioactive compounds for the CVD treatment, and key targets included thrombin, calmodulin, estrogen receptor, Scn1a, HSP90 (Heat Shock Protein 90), PTGS1 (Prostaglandin-Endoperoxide Synthase 1), and PTGS2 proteins. Enrichment analysis revealed that NF-B, VEGF, T cell receptor, calcium, apoptosis, FoxO, HIF1, TNF, and PI3K/Akt were synergistically affected [104]. Bioactive compounds of Qishenkeli, a TCM, using the drug CIPHER-CS could target the renin-angiotensin system (RAAS) and Angiotensin II receptor (AT1R) to treat CHD [105]. Another TCM, without the identification of compounds, targeted NF-*κ*B and TNF signaling pathways to protect against CHD [106]. *Allium macrostemon* Bunge and *Trichosanthes kirilowii* anti-CHD effects were determined based on bioactive compounds such as macrostemonoside d, gitogenin, luteolin, naringenin, and smilagenin. Further experimental analyses proved that the expression of KALM1, PKA (Protein Kinase A), PI3K, and PLCy genes was changed [107]. Anti-CHD effects of a Mongolian herb, namely, *Fructus Choerospondiatis* and *Nutmeg* were via targeting the PI3K-Akt, FoxO, MAPK, and EGFR signaling pathways and lipolysis and leukocyte migration. The main bioactive compounds included gallogen, macelignan, chebulic acid, citric acid, succinic acid, quercetin, and safrole. In vitro and in vivo studies also confirmed the findings [100]. In a study using ML mainly targeting oxidative stress, the main bioactive compounds of HMs were introduced for the healing of insomnia and CVD [108]. *Linum usitatissimum* L. bioactive compounds for the treatment of uncomplicated pelvic inflammatory disease have included polyphenols and flavonoids [109].

## 10. Major Herbal Bioactive Compounds With Potential of CVD/CHD Healing Traits

### 10.1. Campesterol

**Figure 2:**
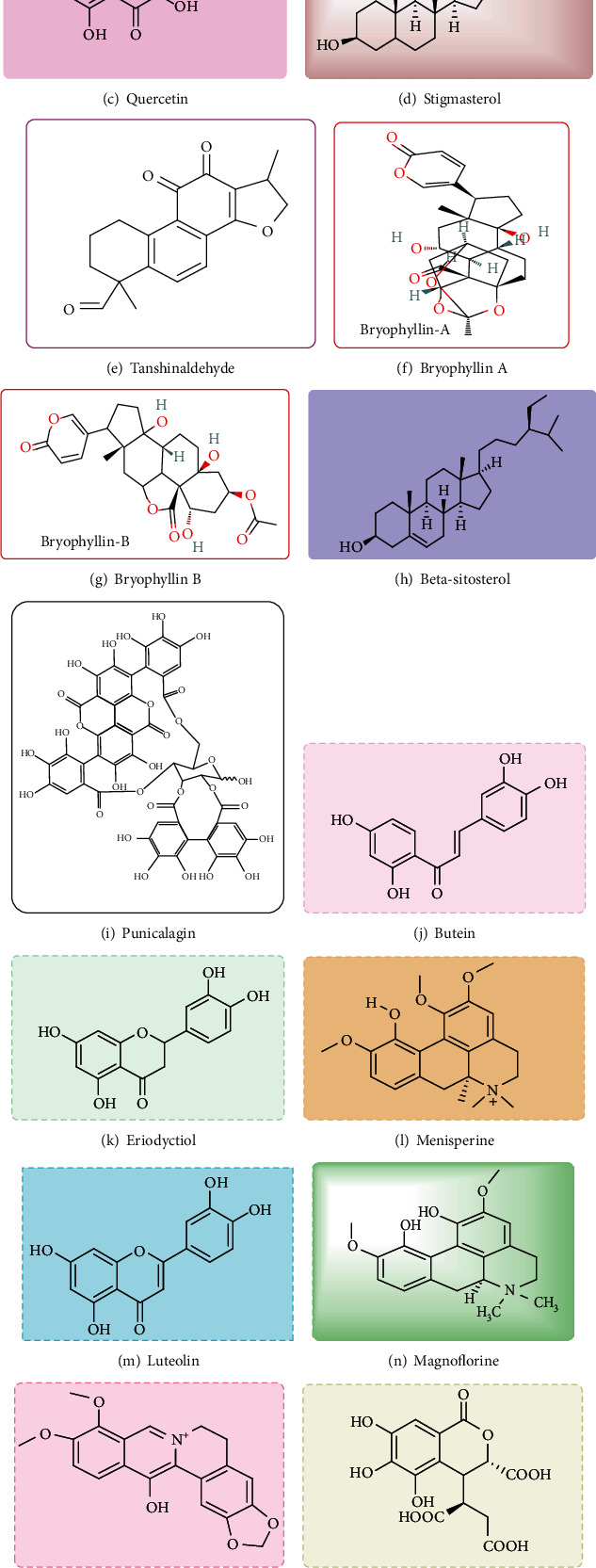
Molecular structure of bioactive compounds in this study.

Campesterol (Figure 2(a)) is a phytosterol found in various vegetables and fruits such as pomegranate, potato, banana, onion, pepper, grapefruit, coffee, cucumber, oat, and lemon grass (citronella) [110]. The chemical structure is similar to cholesterol. In the 1950s, plant sterols exhibited lowering cholesterol and LDL [111]. This molecule is believed to compete with cholesterol, thereby reducing its absorption in the human intestine [112].

### 10.2. Naringenin

Naringenin (Figure 2(b)) is a flavonoid and common flavone in grapefruit and is found in different herbs and fruits such as sour orange, cocoa, tomatoes, bergamot, water mint, tart cherries, and beans [113–115]. It displays good activity for treating various kinds of heart diseases due to its antioxidant and anti-inflammatory effects [116].

### 10.3. Quercetin

Quercetin (Figure 2(c)) is a polyphenol bioactive compound that exists in various fruits, seeds, vegetables, leaves, and red onions. Quercetin decreases systolic blood pressure significantly [117]. This compound outlined good activity against CVD such as hypertension, aging effects, angiotensin-converting enzyme activity, and endothelial-dependent and independent functions [118]. Quercetin, as a polyphenol flavonoid, is naturally present in different vegetables and fruits. One of these bioactive compounds showed potent anticancer properties due to different mechanisms such as cellular signaling and its ability to inhibit enzymes responsible for the activation of carcinogenicity. Quercetin confers anticancer activity via binding to proteins and cellular receptors. Quercetin has a vascular protective effect associated with eNOS upregulation, blood GSH (glutathione) redox ratio, and reduction of oxidative stress. The research showed that it could reduce the risk of CVD.

### 10.4. Stigmasterol

Stigmasterol (Figure 2(d)) is an unsaturated phytosterol found in various herbal essential oils. This bioactive compound exists in calabar bean, soybean, and rape seed. It can also be found in herbalism practices, such as the Chinese herbs *Ophiopogon japonicus* and *Mirabilis jalapa* [119, 120]. It is used as a food additive in different companies in the European Union and the United Kingdom. Percy Lavon Julian was reported as a precursor for the industrial large-scale manufacture of semisynthetic progesterone [121, 122]. It is also used as the precursor of vitamin D3 [122]. However, a study exhibited that stigmasterol may cause cardiac injury, making its consumption cautious.

### 10.5. Tanshinaldehyde

Tanshinaldehyde (Figure 2(e)) is a bioactive and natural product found in *Salvia przewalskii*, with a molecular formula of C_19_H_18_O_4_ and a molecular weight of 310.3 g/moL. It has shown a wide range of activities such as anticardiac hypertrophy, antioxidant, antiarrhythmia, antiatherosclerosis, and antioxidant traits [123].

### 10.6. Bryophyllin A and Bryophyllin B

Bryophyllin B and Bryophyllin A (Figures 2(f) and 2(g)) are bioactive compounds found in *Bryophyllum pinnatum*. These compounds repress the AMPK and iNOS activity, playing a substantial role in the treatment of atherosclerosis [95]. Bryophyllin B has deciphered proper anticancer and anti-inflammatory activities [124]. Bryophyllin A also showed good anticancer activity against various cell lines such as P388, L1210, and human KB, A549, and HCT8 cells [125, 126].

### 10.7. *β*-Sitosterol

*β*-Sitosterol (Figure 2(h)) is a type of herbal sterol with a chemical structure similar to cholesterol. It is found in nuts, avocados, and vegetable oil and is used as a food additive [127]. This bioactive compound is used in veterinary medicine to induce growth in cattle. Also, this compound is one of the most abused anabolic steroids in sports [128, 129].

### 10.8. Punicalagin

Punicalagin (Figure 2(i)) is a phenolic compound found in two forms of alpha and beta in pomegranates (*Punica granatum*), *Terminalia catappa*, *Terminalia myriocarpa* [1], and *Combretum molle*, a plant species found in South Africa [130, 131]. Research showed that punicalagin pretreatment attenuates myocardial ischemia–reperfusion injury via activation of the AMPK enzyme [132].

### 10.9. Butein

Butein (Figure 2(j)) is an *α*,*β*-unsaturated carbonyl compound of the chalconoids [133]. This bioactive compound can be found in *Dahlia*, *Butea*, and *Coreopsis* [134]. It has shown a wide range of biological activities such as antioxidant, anti-inflammatory, hypotensive, anticancer, and aldose reeducates and neuroprotective effects, and glycation end-product inhibitory function [135]. It has a high ability to inhibit the aromatase process in the human body [136].

### 10.10. Eriodyctiol

Eriodyctiol (Figure 2(k)) is a phenolic flavonoid extract from yerba santa which is a plant in North America. Also, it was found in *Eupatorium arnottianum* and *Millettia duchesnei* and in lemons [137, 138]. It has various biological activities such as antioxidant, anti-inflammatory, hypotensive, and antimicrobial; it also has protection against cardiovascular issues and skin protection [139].

### 10.11. Menisperine

Menisperine (Figure 2(l)) iodide is a bioactive compound belonging to the group of alkaloids and is used as an antispasmodic. It has a wide spectrum of biological activities such as anti-inflammatory, antitussive, and anti-inflammatory properties. It has been demonstrated that menisperine could inhibit matrix metalloproteinases (MMPs) by binding to zinc ions. Also, it inhibits the production of acetylcholine at synapses in neurons, leading to its antispasmodic effects [140–142].

### 10.12. Luteolin

Luteolin (3⁣′, 4⁣′, 5⁣′, 7⁣′-tetrahydroxyflavone, Lut) (Figure 2(m)), a yellow crystalline bioactive substance obtained from *Reseda luteola*, is known as a flavone. Luteolin has a broad spectrum of biological activities such as antitumor, antiapoptotic, anti-inflammatory, and antioxidant traits [143].

### 10.13. Magnoflorine

Magnoflorine (Figure 2(n)) is a benzylisoquinoline alkaloid that has been isolated from different kinds of *Menispermaceae* families such as *Cissampelos pareira*, *Pachygone ovata*, and *Sinomenium acutum* [144, 145]. It has demonstrated a wide range of biological activities such as anti-inflammatory [146] and antifungal activities [147]. It has been identified to be an inhibitor of NF-кB activation and to be an agonist at the *β*2-adrenergic receptor [148].

### 10.14. Hydroxyberberine

Hydroxyberberine (Figure 2(o)) is an isoquinoline alkaloid obtained from various medicinal herbs including *Phellodendron amurense* and *Berberis aristata* [149, 150]. This bioactive compound is commonly used in HM. It has outlined a broad range of pharmacological activities such as anti-inflammatory, anticancer, and antibacterial effects [151]. 13-Hydroxyberberine also exerts potential anticancer and antimalarial activities [152].

### 10.15. Chebulic Acid

Chebulic acid (Figure 2(p)) is a phenolic bioactive compound that is isolated from *Terminalia chebula.* It has revealed free radical scavenging and antioxidant activities in vitro [153]. *Terminalia chebula* showed a wide range of biological activities such as antifungal, antibacterial, antiviral, antitumor, and antidiabetic activities [153].

### 10.16. Ellagic Acid

Ellagic acid (gallogen) (Figure 2(q)) is a polyphenol compound found in various vegetables and fruits [154]. It can be prepared from the hydrolysis of tannins including geraniin and ellagitannin. Its therapeutic effects mostly go back to the antioxidant and antiproliferative activities. The research has confirmed the ellagic acid improvement of cardiovascular function, particularly among obese adolescents [155].

Those major herbal bioactive compounds with potential for CVD/CHD healing traits have been depicted in Figures 2(a), 2(b), 2(c), 2(d), 2(e), 2(f), 2(g), 2(h), 2(i), 2(j), 2(k), 2(l), 2(m), 2(n), 2(o), 2(p), and 2(q).

## 11. Future Prospects

Considering the high burden of CVD globally, the poor effects or side effects of current therapy options, and the increasing rate of the disease, prediction and discovery of potential natural compounds are crucial to decreasing the death rates. Computational tools have demonstrated efficiency as rapid, inexpensive, and accurate basic predictions of natural bioactive compounds. These tools should be followed by further confirmatory experimental studies including in vitro, in vivo, and clinical trials to evaluate dose-effect relationships. International plans for development towards the application of novel safe therapies or combination therapies are essential to decreasing CVD as the main cause of global death. Educational plans are helpful to motivate society for physical activity, consumption of healthy foods, and avoidance of risky behaviors. In the future, following personalized medicine using genetic testing and molecular profiling can be promising. Targeted therapies specifically for certain pathways or molecules involved in the disease process will contribute to efficient therapy [14, 156]. Stem cell therapy can also regenerate damaged heart tissue and improve cardiac function [157, 158]. Noninvasive interventions and digital health technologies are promising for disease control. The gene editing technologies, such as CRISPR-Cas9, will open new avenues in therapy. In addition, the development of vaccines or antibodies can play a role in CVD immunotherapy [159]. The biomarkers with the potential to accurately predict the risk of developing CVD will contribute to the prognosis and therapies. The employment of AI and ML and collaboration and interdisciplinary approaches are also paramount in the treatment and control process [160, 161].

## 12. Conclusion

In silico studies have discovered potential anti-CVD and anti-CHD herbal bioactive compounds with efficient interaction with main targets. Network pharmacology and molecular docking have provided theoretical evidence for potential herbal compounds that need experimental work for confirmation. Adoption of compounds based on efficiency and main mechanisms of action also needs confirmatory work. Several in silico studies have been further evaluated by in vitro experiments such as anti-CVD tanshinaldehyde and antiatherosclerosis punicalagin. In silico and in vivo studies have revealed the menisperine and luteolin anti-CHD effects. The healing effects of butin, butein, and eriodyctiol anti-IS effects have been revealed in vitro and in vivo following in silico screening. The mechanisms of action of bioactive compounds in this case include cell signaling and inhibition of inflammation and oxidative damage, decrease of lipid accumulation, and regulation of metabolism and immune cells.

## Figures and Tables

**Table 1:** Major natural bioactive compounds found to treat CVD, CAD, CHD, atherosclerosis, and ischemic stroke using computational studies.

**Bioactive compound**	**Target**	**DS (kcal/moL)**	**Data bank/source**	**In vitro*/*in vivo**	**Disease**	**Reference**
4926615 e-pharmacophore	PDE5A and PDE3A	−12.10 and −10.01	CoCoCo	No/no	CVD	[90]
Campesterol	GSK3B	−12.4	Sea buckthorn	No/no	CAD	[97]
Naringenin	PTGS2 and NOS3	−9.4 and −8.2	HERB, TCMSP, and GeneCards	No/no	CHD	
Quercetin	PTGS2 and NOS3	−9.7 and −8.6				
Quercetin	PTGS2	−9.1	Medicinal herb	No/no	CHD	[93]
Stigmasterol	PTGS2	−9.7				
Tanshinaldehyde	PIK3CA	−11.1	Medicinal herb	Yes/no	CVD	[98]
Bryophyllin A, Bryophyllin B	AMAPK	−9.3 and −9.9	Medicinal herb	No/no	Atherosclerosis	[95]
Quercetin	AKT1 and MAPK1	−6.51 and −6.53	Medicinal herb	No/no	CHD	[82]
Beta-sitosterol		−7.8 and −7.5				
Punicalagin	CD36 and TLR4	−9.3 and −9.0	Medicinal herb	Yes/no	Atherosclerosis	[26]
Butein	AKT1	−10	Medicinal herb	Yes/yes	IS	[99]
Eriodyctiol	AKT1	−10				
Butin	AKT1	−10				
Luteolin, kaempferol	TNF	−9 and −8.9	Medicinal herb	No/no	CHD	[96]
Triptofordin B2, hypodiolide A, celaxanthin, and celallocinnine	Akt, APP, MAPK1, PIK3CA, and TP53	—	Medicinal herb	No/no	CVD	
Menisperine, luteolin, magnoflorine, 13-hydroxyberberine^a^	MAPK1, AKT1, SRC, and EGFR	—	Medicinal herb	No/yes	CHD	[82]
Gallogen (ellagic acid) and chebulic acid^a^	PI3K-Akt, FoxO, MAPK, and EGFR	—	Medicinal herb	Yes/yes	CHD	[100]

Abbreviations: AMAPK, adenosine monophosphate-activated protein kinase; CAD, coronary artery disease; CHD, coronary heart disease; CoCoCo, commercial compound collection; CVD, cardiovascular disease; DS, docking score; GSK3B, glycogen synthase kinase-3 beta; IS, ischemic stroke; PDE3A, phosphodiesterase-3; PDE5A, phosphodiesterase-5; TwH, *Tripterygium wilfordii* Hook F.

^a^Network pharmacology.

## Data Availability

The data that support the findings of this study are available upon reasonable request from the corresponding author.
